# Biodegradable Amphoteric Surfactants in Titration-Ultrasound Formulation of Oil-in-Water Nanoemulsions: Rational Design, Development, and Kinetic Stability

**DOI:** 10.3390/ijms222111776

**Published:** 2021-10-29

**Authors:** Ewelina Waglewska, Urszula Bazylińska

**Affiliations:** Department of Physical and Quantum Chemistry, Faculty of Chemistry, Wroclaw University of Science and Technology, Wybrzeze Wyspianskiego 27, 50-370 Wroclaw, Poland; ewelina.waglewska@pwr.edu.pl

**Keywords:** nanoformulations, betaine-type surfactants, mild surfactants, ternary phase diagrams, kinetic stability, backscattered profiles, cocamidopropyl betaine, coco-betaine

## Abstract

Amphoteric amphiphilic compounds, due to their unique properties, may represent a group of safe and biocompatible surface-active agents for effective colloidal stabilization of nanoformulations. For this reason, the aim of this work was to develop and characterize the oil-in-water nanoemulsions based on two betaine-derived surfactants with high biodegradability, i.e., cocamidopropyl betaine and coco-betaine. In the first step, we investigated ternary phase diagrams of surfactant-oil-water systems containing different weight ratios of surfactant and oil, as the betaine-type surfactant entity (S), linoleic acid, or oleic acid as the oil phase (O), and the aqueous phase (W) using the titration-ultrasound approach. All the received nanoemulsion systems were then characterized upon droplets size (dynamic light scattering), surface charge (electrophoretic light scattering), and morphology (transmission electron as well as atomic force microscopy). Thermal and spinning tests revealed the most stable compositions, which were subjected to further kinetic stability analysis, including turbidimetric evaluation. Finally, the backscattering profiles revealed the most promising candidate with a size <200 nm for potential delivery of active agents in the future cosmetic, pharmaceutical, and biomedical applications.

## 1. Introduction

Over recent years, the application of various colloidal formulations (i.e., liposomes, solid lipid nanoparticles, micelles, nanoemulsions, nanocapsules, or nanospheres) has attracted considerable interest in the field of nanomedicine, drug delivery, as well as pharmaceutical and cosmeceutical technology [1,2,3,4,5,6]. By carefully designing them and selecting an appropriate preparation method and structure-building components, it is possible to obtain nanocarriers with extended circulation time in the bloodstream, which will provide their cargo to the target site. Considering this, it has become very significant for conducting intensive research to develop novel and safe dispersion systems, whose appropriate kinetic stability and versatility will be able to improve the bioavailability of many drugs, by reducing their side effects as well as provide maximum application benefits, i.e., anti-aging, therapeutic, diagnostic, or conditioning [7].

Nanoemulsions (NEs) (also defined as submicron emulsions, ultrafine emulsions, or miniemulsions) are nano-sized (20–500 nm) droplets emulsions that have gained popularity as safe and efficient nanostructured formulations in droplet engineering due to their increased surface-to-volume ratio, smaller particle size, and greater mobility, as well as enhanced stability and protection against premature degradation [1,8]. They are transparent or translucent isotropic dispersions consisting of two immiscible water and oil phases. One of these liquids is dispersed in the other through a suitable surfactant mixture, forming a colloidal nanosystem [9]. Due to the possibly identical droplet size and subsequently numerous inaccuracies in the scientific literature, NEs are often confused with thermodynamically stable microemulsions (10–100 nm). Therefore, the most considerable difference between these colloidal systems is precisely their stability and droplet size range. NEs, compared to microemulsions, are thermodynamically unstable systems. However, microemulsion stability requires the use of a high surfactant concentration (typically above 20%) and/or the addition of a co-surfactant, which in turn causes high system toxicity, and hence the advantage of nanoemulsions over microemulsions. Furthermore, NEs are relatively less sensitive to physical and chemical changes due to their high kinetic stability. Consequently, these formulations are widely used in different fields, such as drug delivery systems, food, cosmetic, or pharmaceutical industries [10]. The selection of relevant oils for the oil phase is a substantial part of the NEs manufacturing. Unsaturated and saturated fatty acids or fatty acid esters improve the therapeutic effect of particular drugs, so they are most needed to be used as an oil phase [11]. NEs are typically divided into oil-in-water (o/w) and water-in-oil (w/o) formulations which can be produced through various approaches, including high-energy and low-energy methods [12,13]. The most common high-energy methods are homogenization, microfluidization, and ultrasonication. For the second group of NEs preparation called low-energy methods (spontaneous emulsification and the phase inversion technique), the physiological properties of the system are used without significant energy consumption. Thus, there is no need for an external force in the form of mechanical devices. However, the stability of the nanosystem obtained in this way can be not efficient. Consequently, the NE fabrication method is critical to the final product, so its selection depends on the formulation’s composition and physicochemical properties required [10,14].

High kinetic stability, small droplet size, and high solubilization capacity make NEs an important nanoplatform to significantly boost the solubility and action effectiveness of poorly water-soluble compounds [15,16,17]. Nevertheless, this dispersion system is thermodynamically unstable, which in turn leads to the occurrence of many different physicochemical phenomena such as creaming, flocculation, coalescence, Oswald ripening, and phase inversion [18]. Thus, its stability can be improved by adding specific emulsifiers, generally surfactant molecules containing both hydrophilic and hydrophobic parts. Due to their amphiphilic structure, surfactants adsorb onto the oil–water interfaces reducing the interfacial tension between these immiscible liquids. Numerous surfactant types are available to create and stabilize NEs, which are divided mainly based on the charge of the polar group of these molecules. Among these are anionic, cationic, nonionic, as well as zwitterionic (amphoteric) compounds. Due to their different properties, it is hugely important to choose the most appropriate one for a particular application [16].

Amphoteric, the so-called zwitterionic surfactants have a negatively as well as a positively charged hydrophilic center and a long lipophilic hydrocarbon chain. They have unique advantages, including high surface activity, high foam stability, low toxicity and irritating, high water solubility, low critical micelle concentration (CMC), and high biodegradability. Because of these properties, amphoteric surfactants are potential candidates for replacing purely ionic stabilizers in a wide range of products [19]. Zwitterionic surfactants like semi-synthetic betaines are used as foam stabilizers in commercial, personal care products due to their gentleness to the skin that is not affected by water hardness and their producing excellent foam with good stability [20]. Coco betaine and cocamidopropyl betaine are examples of amphoteric biobased surfactants produced from natural “vegan” friendly raw materials such as coconut oil [21]. Additionally, these carboxybetaines are suitable for biological applications due to the lack of total charge that weakens their interactions with proteins [22,23]. Thus, the betaines application as effective biocompatible stabilizers for the formation of kinetically stable NEs could be advantageous and competing with other ionic and nonionic nanoformulations.

**Figure 1:**
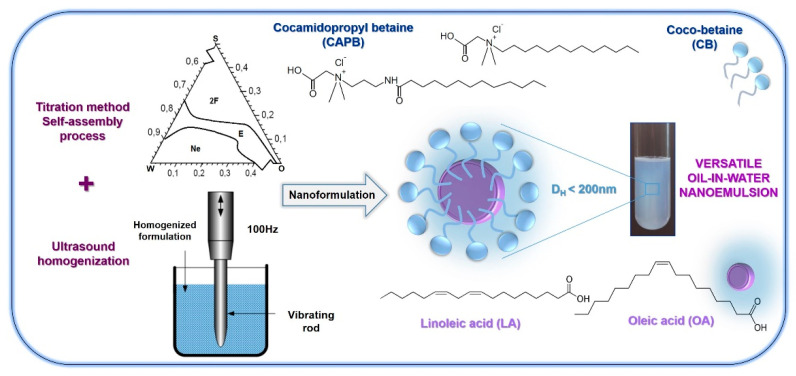
The general approach to the instigated studies.

The physicochemical properties and biocompatibility of NEs may depend on various parameters, including the concentration of surfactants/emulsifying agents as well as oil phase, their properties of the liquid/liquid interface, and also surfactant-to-oil ratio. As a result, the composition of the NEs should be carefully selected to obtain stable nanosystems. Thus, the principal objective of the present contribution (see Figure 1) was to examine the effect of commercially available biodegradable amphoteric surfactants—very mild to the skin and mucous membranes, i.e., cocamidopropyl betaine (CAPB) and coco-betaine (CB), in the fabrication of kinetically stable oil-in-water (o/w) nanoemulsions by using complex titration-ultrasound cavitation approach. The oil phases were selected from the group of naturally occurring unsaturated omega-6 and omega-9 fatty acids, such as linoleic acid (LA) and oleic acid (OA). Initially, the phase behavior (ternary phase diagrams) of surfactant–oil–water (S:O:W) systems containing various ratios of the selected surfactant (S), oil (O), and water phase (W) was examined. In the next step, the influence of various preparation parameters, such as the type of surfactant, oil composition, and surfactant-to-oil ratio on the characteristics of the received nanosystems (i.e., droplet size and charge) was investigated. Finally, the kinetic stability evaluation provides the selection of the most optimal, long-term, and versatile colloidal formulation for further cosmetic, biomedical, and pharmaceutical applications. To the best of our knowledge, the presented formulations are the first example of environmentally friendly nanoemulsions stabilized by amphoteric cocamidopropyl and coco-betaine with oleic unsaturated acids droplets obtained by the complex titration-ultrasound method.

## 2. Results and Discussion

In the proposed research, we have designed and developed a new long-term colloidal oil-in-water (o/w) nanoemulsion system, stabilized with very mild amphoteric surfactants with high biodegradability, i.e., cocamidopropyl betaine (CAPB) and coco-betaine (CB). An additional benefit is the use of naturally occurring omega-6 and omega-9 unsaturated fatty acids, such as linoleic acid (LA) and oleic acid (OA), which can be found naturally in “good” oils such as olive oil, safflower oil, or avocado oil, as well as nuts and seeds, as the oil phase. Furthermore, the amphoteric nanoemulsions were formulated by the complex aqueous self-assembly titration and ultrasound homogenization methods as a result of phase transitions supported by an external force produced during the emulsification process (Figure 1). This rationally designed methodology is a joining of sustainable approach belonging to low-energy emulsification techniques making use of the chemical energy stored in the components, with the simplicity and time saving of ultrasound high-energy.

### 2.1. Evaluation of Phase Diagrams

The construction of phase diagrams is crucial to assess the phase behavior of oil–water systems stabilized by surface-active agents. The diagrams for three-component mixtures, the so-called Gibbs phase triangles, deliver information about different phase boundaries as a function of the system composition variables. Moreover, it is also possible to deduce the structural organization of the surfactant aggregates allowing the comparison of their diverse efficiency for a specified application [24]. A formulation type, i.e., nano or macroemulsion (emulsion) of known composition, can be easily accessed via visual observation of the identified boundaries of a one-phase region. Most often, the primary stabilization of the system occurs in the bulk aqueous phase or on the droplets interface, depending on the chemical nature of the involved components. The role of the surfactant is to lower the surface tension by preferential adsorption at the oil/water interface, providing a mechanical barrier to coalescence, flocculation, or other destabilization processes of a given formulation. Thus, the selection of appropriate emulsifiers is essential in controlling the functional properties of nanoemulsions, including surface charge, droplet size, their polydispersity, and their long-term (kinetic) stability [1,4,9,25].

**Figure 2:**
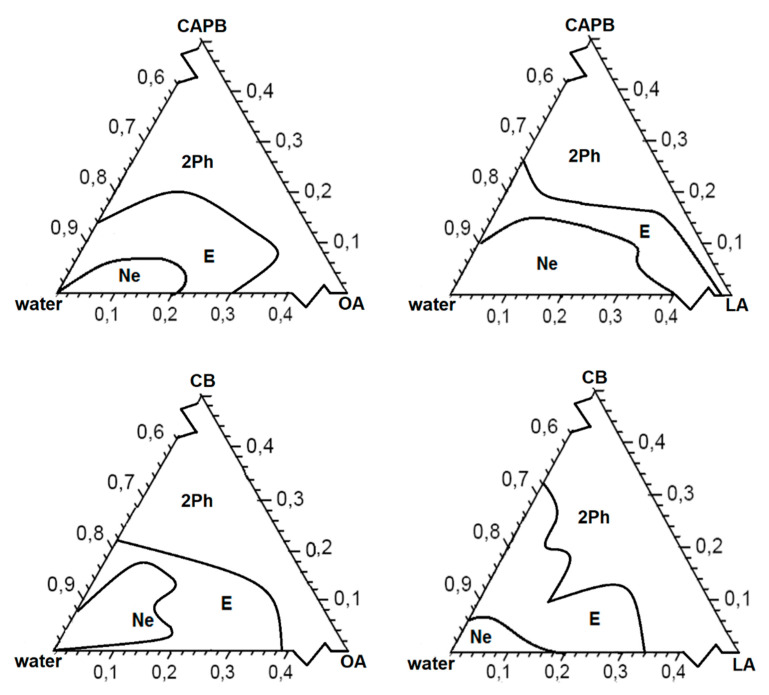
Partial phase diagrams of the obtained formulations stabilized with amphoteric betaine-type surfactants. Ne—oil-in-water type nanoemulsion (o/w), E—emulsion, 2Ph—double phases.

In our research, the partial phase diagrams of the formulations containing oils (OA or LA), water, and amphoteric surfactants (CAPB or CB) are shown in Figure 2. The aqueous phase titration followed by ultrasound homogenization was done for each weight ratio of oil and surfactant (1:9, 2:8, 3:7, 4:6, 5:5, 6:4, 7:3, 8:2, and 9:1). The visual observation was carried out for semi-transparent, opalescent, bluish oil-in-water (o/w) nanoemulsion (NE) and milky, creamy emulsion (E). 

The physical state of the NEs was marked on a three-component phase diagram with one axis representing aqueous phase (W), the other showing oil (OA or LA), and the third representing the betaine-type surfactant (CB or CAPB). In all cases, the NE and E area was present when at least 40% W phase was added to the system. Therefore, for better visualization of the formulation areas, partial diagrams were used. Finally, twelve different formulations were selected with the same S:O compositions (Table 1) from the o/w NE region for each partial phase diagram constructed. These NE systems were then characterized upon droplets size (D_H_), polydispersity (PdI), ζ-potential, and morphology (TEM and AFM).

### 2.2. Estimation of the Droplet Size, Surface Charge, and Morphology

The droplet size and polydispersity are crucial factors to identify the type of the obtained formulation as well as to understand its behavior. Furthermore, in addition to composition, the final application (cosmetic, pharmaceutical, or biomedical) of the designed system is also influenced by the droplet size and size distribution [1,4,6]. From Table 1, it can be seen that formulations with a lower concentration of the components, i.e., surfactant and oil phases, revealed the smallest NE droplets (D_H_, 65–78 nm and PdI < 0.3 for 1% S/0.5% O composition). This correlation was observed with both types of surfactants and oils. As the concentration of the internal phase components increased, the droplet size and its dispersity increased too. The most unimodal size and size distribution were observed for NEs stabilized by CAPB with the S:O:W ratio equal to 3:1:96 (system 4 and system 10). In the case of system 6 with 5% LA/2% CAPB, the droplet size exceeded 300 nm, but the PdI did not exceed 0.3. However, the larger droplet size may make such a system more susceptible to nanoemulsion destabilization processes such as flocculation, coalescence, sedimentation, or creaming [9,11]. Thus, only the most unimodal nanoformulations (darkened lines in Table 1) with the optimized composition and size parameters (D_H_ 100–300 nm, PdI < 0.3) were selected for further dispersion stability investigations.

Considering the efficacy of the resulting dispersions in terms of their usefulness in cosmetic, pharmaceutical, and biomedical applications, one more important characterizing parameter should be taken into account to evaluate the NE stability, namely their zeta potential (ζ-potential) values. The ζ-potential evaluation by electrophoretic light scattering (ELS) is an efficient tool to estimate the droplet surface charge, which can be employed to understand the formulation’s physical stability. Generally, a large positive or negative value of ζ-potential indicates a good physical stability of the formulations due to the electrostatic repulsion of individual droplets. It has been proven that long-term stable NEs characterized by a high value of the ζ-potential are effectively stabilized by electrostatic repulsion. When this potential is low (0 to ±5 mV), the attraction of the dispersion droplets exceeds their repulsion, and the nanoemulsion is rapidly destabilized by processes such as coagulation and flocculation [1,9].

Thus, it is necessary to assess the ζ-potential magnitude of the studied formulations to identify the charge of oil droplets in the NE systems and to predict their stability. The ζ-potential values of the studied NEs (Table 1) were in the range from −7 to −29 mV suggesting that stable dispersions were obtained. As expected, these values were dependent on the concentration of the applied amphoteric surfactants. Generally, in formulations with a lower concentration of surfactants, the zeta potential values were the highest from −14 to −29 mV. The observed phenomenon is in a good correlation with the earliest studies on the zeta potential behavior of betaine-type surfactants [26]. Although these nanoemulsions showed potentially higher stability, their too small droplet size (65–78 nm) for cosmetic and pharmaceutical applications [1], made it possible to select formulations with optimally better physicochemical parameters in terms of size, polydispersity, and stability for further experiments (Table 1).

**Figure 3:**
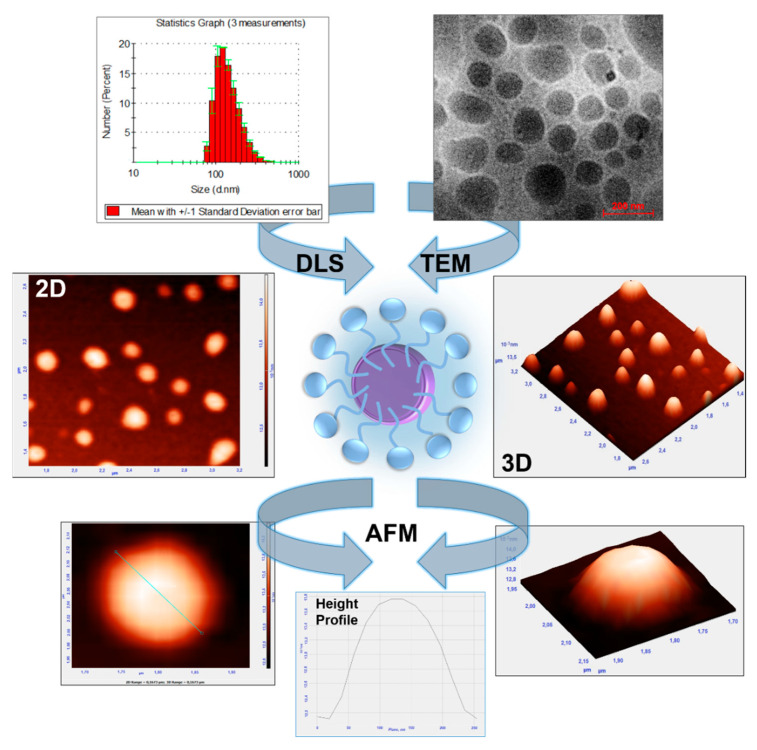
Size distribution (DLS) as well as morphology imaging via transmission electron microscopy (TEM) and atomic force microscopy (AFM) of the optimized nanoemulsion system (example for sample 10, see the sample descriptions in Table 1).

Microscopic imaging techniques allow the delivery of the most consistent data on morphology, i.e., size, shape, polydispersity, and even the chemical composition of the produced nanoobjects. Accordingly, the studied nanoemulsions were first characterized by transmission electron microscopy (TEM), a powerful quick method for revealing the structure of nanocolloid systems with very high resolution, the limit of which in the case of the best microscopes can even be 0.15 nm. As it was shown by Lewinska’s group [4,25], TEM was effectively applied for imaging of “soft” colloidal formulations as nanoemulsions with good resolution, contours, and contrast of images. In our studies, the typical image of the obtained NE stabilized by an amphoteric betaine-type surfactant is presented in Figure 3. The imaging showed the presence of near-spherical nanostructures with a more or less uniform size around 150 nm and good droplet distribution. The additional imaging technique involves the application of easily available atomic force microscopy (AFM). In addition to imaging nanoobjects, AFM allows for the determination of the size profiles of individual nanodroplets. The 2D and 3D images obtained with the tapping can also show nearly spherical and more regular-shaped objects, demonstrating the precise and successful acquisition of the NE droplet. Furthermore, the images revealed that the NE droplets were well separated indicating their effective stabilization by the surrounding surfactants. The size of the nanostructures confirms the results obtained by dynamic light scattering, also shown in Figure 3.

### 2.3. Kinetic Stability Assessment 

Seven different formulations from the o/w NE region (Table 1) with the most favorable physicochemical parameters, i.e., hydrodynamic diameter 100–300 nm and polydispersity index below 0.3, were chosen to provide the next step of our studies, i.e., dispersion stability tests according to the experimental procedure. The thermal and spinning stability of the selected ternary systems was observed for 72 h during six cycles between the refrigerator (4 °C) and room temperature (25 °C). Subsequently, those nanoemulsions that did not show any phase separations were centrifuged at 4000 rpm for 30 min. Finally, six of the most stable systems which showed no phase separation, creaming, coalescence, or phase inversion upon these tests were subsequently selected for turbidimetric analysis by using backscattering profiles.

**Figure 4:**
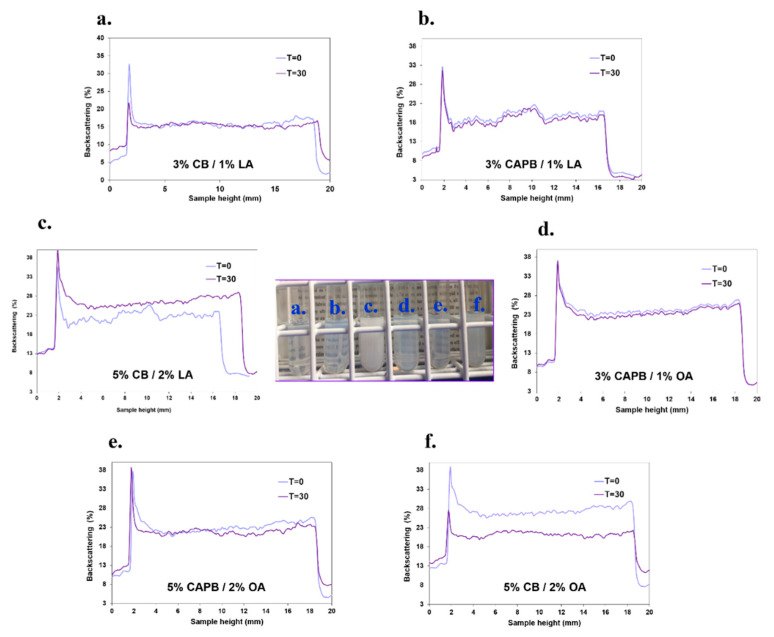
Backscattered profiles (BS) along the nanoemulsion as a function of the sample height (mm), analyzed for freshly prepared formulations (T = 0) and those stored for 30 days. The image presents the studied NE: sample 1 (**a**), sample 4 (**b**), sample 3 (**c**), sample 10 (**d**), sample 12 (**e**), sample 9 (**f**).

The main principle of turbidimetric measurement is based on the migration and size change of particles/droplets, which cause the backscattering signals to diverge in time. From the characteristics of the actual NEs condition analysis performed with the Turbiscan Lab, curves showing the percentage of BS as functions of the measurement chamber height (expressed in mm) were obtained. By analyzing the distance between the curves in the BS profiles of the NEs at time 0 (freshly prepared) and after 30 days of storage at room temperature, it was possible to determine the dynamics of the destabilization processes occurring in individual samples (Figure 4). Based on the graphs shown in Figure 4, we conclude that the formulations stabilized by cocamidopropyl betaine show a high colloidal stability, as demonstrated by the lack of significant changes in these samples after 30 days of storage. As the length of the surfactant tail increases, the interfacial stability of the film increases, which is related to the presence of strong hydrophobic interactions between the hydrocarbon chains of the surfactant [27]. Therefore, the longer hydrocarbon chain in the CAPB structure compared to CB allowed for a more stable formulation. This longer hydrocarbon chain may also be related to the zeta potential value of the analyzed samples. Investigating the backscattering lines presented in Figure 4, we could observe an influence of the zeta potential value on the stability of the samples. Those with the lowest zeta potential, i.e., sample 7 shown in Figure 4c (zeta potential of −7 mV) and sample 9 shown in Figure 4f (zeta potential of −10 mV), show the most significant changes in BS profiles after 30 days of their storage. Both samples were also stabilized by coco-betaine, and additionally, the components (S and O) concentration was the highest. Consequently, the stability of the nanosystems also depends on the concentration of surfactant and oil phases. As the concentration of these components increases, rapid destabilization phenomena are observed, demonstrated by visible changes in backscattering, which is particularly evident for CB-stabilized formulations. The backscattering increases versus time at the bottom of the sample containing 2% LA and 5% CB (left side of the spectrum) indicating the occurrence of sedimentation. In the same formulation, the shift on the right side of the profile (top of the sample) corresponding to day 30 is due to the creaming of the preparation stabilized with 5% CB (Figure 4c). However, for the nanoformulation containing 5% CB and 2% OA, a decrease in backscattering in the lower region, which can be triggered by a decrease in the concentration of dispersed particles in the sample medium, suggests the occurrence of a clarification process. In addition, it follows from this graph that the decrease in backscattering after 30 days of storage in the middle part of the sample indicates the emergence of the coalescence process (Figure 4f). In this case, the amount of surfactant in the formulation was high enough, which probably caused the formation of aggregates in the aqueous phase. Coating the oil surface with 3% surfactant was sufficient to prevent oil droplets from approaching each other, so no coalescence was observed in the formulations (Figure 1a). When the concentration of surfactants used was increased to 5%, less stable NEs were obtained. This phenomenon also observed during the DLS measurements, likely led to a decrease in the surfactant concentration available to coat the oil droplets, ultimately causing an increase in the NE droplet size, leading to the coalescence phenomenon [28]. The obtained results showed that the overlap of the individual curves indicates a very high stability and slow rate of the destabilization process for the analyzed formulation stabilized by CAPB. Consequently, the o/w NEs containing amphoteric cocamidopropyl betaine surfactant can serve as a promising nanoplatform to prepare novel nanoemulsion-based delivery systems for future biological applications.

## 3. Materials and Methods

### 3.1. Chemical Reagents 

The amphoteric surfactants (cocamidopropyl betaine (CAPB) and coco-betaine (CB)) were a kind gift from PCC Exol Rokita (Brzeg Dolny, Poland). The oleic phases (oleic acid (OA) and linoleic acid (LA)) were purchased from Avantor Performance Materials Poland S.A. (formerly POCH S.A., Gliwice, Poland). Water used for all experiments was doubly distilled and purified by means of a Millipore (Bedford, MA, USA) Milli-Q purification system.

### 3.2. Preparation of Oil-in-Water Nanoemulsions by Titration-Ultrasound Approach 

Ternary phase diagrams composed of S—surfactant (CAPB or CB), O—oil (OA or LA), and W—water, i.e., S:O:W systems were prepared via a composed aqueous titration-ultrasound method to examine the phase behavior of the samples. The phase boundaries were determined by the stepwise addition of water to the weighted mixture of the S:O:W components. After the addition of each portion of water, the samples were sonicated by a tip sonicator (UP100H ultrasonic processor developed by Hielscher Ultrasonics GmbH, Teltow, Germany; cycle, 0.9; amplitude, 80%) for 2 min and kept in thermostated baths at 25 °C to equilibrate. The amount of aqueous phase added was varied to obtain a water concentration ranging from 5% to 99% of the total volume in about 5% intervals. The boundary lines drawn on the phase diagram lie at equal distances between consecutive experimental measurements on both sides of the phase boundary. Visual observation was made after each 5% addition of the aqueous phase to the S:O mixture and its equilibration. Based on the visual assessment, the obtained formulations were categorized as follows: (i) transparent and translucent with a blue sheen: oil-in-water (o/w) nanoemulsion (NE); (ii) milky or cloudy: emulsion (E); (iii) two separate phases (2Ph). Selected formulations were subjected to further physicochemical characterization.

### 3.3. Physicochemical Characteristics of the Obtained Nanoformulations

#### 3.3.1. Particle Size and Polydispersity Index by Dynamic Light Scattering

The measurements of the average particle size (D_H_) and polydispersity index (PdI) values of the nanoemulsion droplets were determined using dynamic light scattering (DLS) method. A Zetasizer Nano ZS (Malvern Instruments, Worcestershire, UK) equipped with a detection angle of 173°, a He-Ne laser (632.8 nm), and an ALV 5000 multibit, multitap autocorrelator (Malvern Instruments, Worcestershire, UK) was used for the analysis. All the measurements were taken at 25 °C in optically homogeneous square polystyrene cells with 4 mL volume. The nanoemulsions were prepared via the above-described titration-ultrasound method and selected sample compositions (indicated in Table 1) were measured without prior dilution process. Each value was calculated as the average of three subsequent instrument runs with at least 20 runs. The DTS (Nano) instrument software was applied for data evaluation.

#### 3.3.2. Zeta Potential by Electrophoretic Light Scattering

The analysis of the nanodroplets charge was provided by zeta (ζ) potential evaluation using electrophoretic light scattering (ELS). A Zetasizer Nano ZS (Malvern Instruments, Worcestershire, UK) was used for the ELS measurements. The applied field strength was 20 V/cm. The results are given as an average of 3 measurements, each with at least 20 runs. All the measurements were determined at 25 °C. The DTS (Nano) program was applied for data evaluation.

#### 3.3.3. Shape and Morphology by Transmission Electron and Atomic Force Microscopies 

*Atomic force microscopy*: the AFM morphology observations were provided by a Veeco NanoScope Dimension V microscope (Plainview, New York, NY, USA) equipped with an RT ESP Veeco tube scanner. The scanning speed was 0.5 Hz, and a low-resonance-frequency pyramidal silicon cantilever resonating at 250–331 kHz was employed at a constant force of 20–80 N/m. The nanoemulsions were prepared via the above-described titration-ultrasound method and next were diluted 20 times prior to the sample specimens’ preparation. Before observations, the nanoemulsions were allowed to adsorb on a fresh mica surface for 24 h by dipping it in the prepared samples. Then, the surfaces were rinsed in double-distilled water and dried at room temperature for about 2 h.

*Transmission electron microscopy*: the TEM imaging was studied by using a FEI Tecnai G2 20 X-TWIN microscope (FEI, Hillsboro, OR, USA). The NE morphology was determined by counting the size of approximately 250 droplets from several TEM images obtained from different grid sites. The nanoemulsions were prepared via the above-described titration-ultrasound method and next were diluted 20 times (similar to AFM), prior to the sample specimens’ preparation. The dispersion sample with volume about 20 µL was placed on a perforated, carbon-film-coated copper grid and stained with 2% uranyl acetate before shooting. NE morphology was determined by counting the size of approximately 250 droplets from several TEM images obtained from different grid sites.

#### 3.3.4. Dispersion Stability Studies

*Thermal and spinning tests*: the analysis was carried out in order to preselect the most stable formulations, which were subjected to three dispersion stability assessments: centrifugation, heating, and cooling cycles, and freeze-thaw cycles according to the previously described protocol [29]. The most stable compositions that did not show phase separation, creaming, coalescence, or phase inversion in these tests were selected for further kinetic stability analysis involving turbidimetric evaluation.

*Turbidimetric tests*: the kinetic stability of the optimized NEs was provided in a cylindrical glass cell at 25 °C by measuring the backscattering (BS) of pulsed near-infrared light (λ = 880 nm). A TurbiScanLab Expert instrument (Formulaction SA, Toulouse, France) able to detect the destabilization phenomena in a few hours was used for the measurements. BS profiles as a function of sample height were collected and analyzed by the instrument software program (Turbisoft, version 2.2.0.82, Toulouse, France). The measurements were performed for freshly prepared NEs, and the same samples stored for 30 days at 25 °C.

## 4. Conclusions

According to the presented results, we have successfully prepared novel long-term oil-in-water nanoemulsions stabilized with biodegradable amphoteric surfactants, i.e., cocamidopropyl betaine and coco-betaine, via the complex titration-ultrasound homogenization approach. Based on the conducted experiments, we can conclude that the most promising nanoformulation was created by cocamidopropyl betaine with a S:O:W weight ratio of 3:1:96. This ternary phase system was characterized by nanometric droplet size (D_H_ < 200 nm), monodisperse size distribution (PdI < 0.15), and negative surface charge (ζ ~ −20 mV). Furthermore, its kinetic stability was confirmed by evaluating the backscattering profiles, which showed no significant changes, meaning that no macroscopic or particle size changes occurred during the analyzed storage periods. Our results indicate that betaine-stabilized nanoemulsions, primarily containing cocamidopropyl betaine, represent a promising versatile nanoplatform for the potential delivery of hydrophobic active compounds in many possible applications, such as cosmetics, pharmaceutics, anticancer treatments, and diagnostics.

## Figures and Tables

**Table 1:** Sample results from DLS and ELS for the obtained nanoformulations.

No.	Composition			D_H_^g^ (d.nm)	PdI ^h^	ζ ^i^ (mV)
1	LA ^a^	CB ^c^	3% S ^e^/1% O ^f^	132 ± 6	0.193 ± 0.02	−12 ± 1
2	LA	CB	1% S/0.5% O	70 ± 3	0.046 ± 0.01	−24 ± 3
3	LA	CB	5% S/2% O	295 ± 8	0.198 ± 0.03	−7 ± 1
4	LA	CAPB ^d^	3% S/1% O	130 ± 6	0.135 ± 0.02	−18 ± 2
5	LA	CAPB	1% S/0.5% O	69 ± 3	0.254 ± 0.03	−29 ± 3
6	LA	CAPB	5% S/2% O	316 ± 9	0.290 ± 0.04	−11 ± 2
7	OA ^b^	CB	3% S/1% O	105 ± 5	0.294 ± 0.04	−17 ± 2
8	OA	CB	1% S/0.5% O	65 ± 2	0.046 ± 0.01	−26 ± 3
9	OA	CB	5% S/2% O	272 ± 8	0.249 ± 0.04	−10 ± 1
10	OA	CAPB	3% S/1% O	165 ± 7	0.148 ± 0.03	−21 ± 3
11	OA	CAPB	1% S/0.5% O	78 ± 4	0.245 ± 0.03	−27 ± 3
12	OA	CAPB	5% S/2% O	183 ± 6	0.161 ± 0.02	−14 ± 2

^a^ LA: linoleic acid; ^b^ OA: oleic acid; ^c^ CB: coco-betaine; ^d^ CAPB: cocamidopropyl betaine; ^e^ S: surfactant; ^f^ O: oil; ^g^ D_H_: hydrodynamic diameter (Z-Ave); ^h^ PdI: polydispersity index; ^i^ ζ: zeta potential, mean ± SD, n = 3. Gray lines: nanoemulsions selected for further stability investigations.
